# Clinical Trajectory and Risk Stratification for Heart Failure with Preserved Ejection Fraction in a Real-World Cohort of Patients with Suspected Coronary Artery Disease

**DOI:** 10.3390/jcm13072092

**Published:** 2024-04-03

**Authors:** Guglielmo Gioia, Karl-Patrik Kresoja, Sebastian Rosch, Anne Schöber, Elias Harnisch, Maximilian von Roeder, Markus Scholz, Sylvia Henger, Berend Isermann, Holger Thiele, Philipp Lurz, Karl-Philipp Rommel

**Affiliations:** 1 Department of Medicine/Cardiology, Heart Center at University Leipzig and Leipzig Heart Science, Strümpellstraße 39, 04289 Leipzig, Germany; guglielmo.gioia@medizin.uni-leipzig.de (G.G.); kresojak@uni-mainz.de (K.-P.K.); sebastian.rosch92@gmail.com (S.R.); elias.harnisch@web.de (E.H.); maximilian.vonroeder@medizin.uni-leipzig.de (M.v.R.);; 2 LIFE—Leipzig Research Center for Civilization Diseases, University of Leipzig, 04109 Leipzig, Germany; markus.scholz@imise.uni-leipzig.de; 3 Institute of Medical Informatics, Statistic and Epidemiology, University of Leipzig, 04109 Leipzig, Germany; sylvia.henger@imise.uni-leipzig.de; 4 Institute of Laboratory Medicine, Clinical Chemistry and Molecular Diagnostics, University Hospital Leipzig, 04103 Leipzig, Germany; berend.isermann@medizin.uni-leipzig.de; 5 Department of Cardiology, University Hospital Mainz, 55131 Mainz, Germany; lurzphil@uni-mainz.de; 6 Cardiovascular Research Foundation, New York, NY 10019, USA

**Keywords:** heart failure with preserved ejection fraction, H_2_FPEF score, coronary artery disease, heart failure hospitalization

## Abstract

**Background**: Heart failure with preserved ejection fraction (HFpEF) is a widespread condition with significant morbidity and mortality. Its clinical heterogeneity may delay the diagnosis. **Aim**: To identify predictors of HFpEF-related hospitalizations in ambulatory patients presenting with elevated cardiovascular risk, suspected coronary artery diseases (CADs), and positive HFpEF screenings. **Methods**: Consecutive patients presenting with suspected CAD, enrolled in the observational LIFE-Heart study (2006–2014, NCT00497887), and meeting HFpEF criteria per the 2016 European Society of Cardiology (ESC) guidelines were categorized according to the presence of “overlapping conditions” potentially masking or contributing to their symptoms. Additional stratification using the H_2_FPEF score (<2: low risk, 2–5: intermediate risk, and ≥6 high risk) was performed. Follow-up for hospitalizations, reasons of hospitalization, and death spanned a median of 6 years. **Results**: Of 1054 patients (66 ± 10 years, 60% male, NT-pro-BNP 286, IQR 183–574 pg/mL), 53% had overlapping conditions, while 47% had “isolated HFpEF”. The H_2_FPEF scores classified 23%, 57%, and 20% as low-, intermediate-, and high-risk, respectively, with consistent proportions across patients with and without overlapping conditions (*p* = 0.91). During the follow-up observational phase, 54% were rehospitalized, 22% experienced heart failure (HF) rehospitalizations, and 11% of patients died. Multivariable logistic regression revealed a high-risk H_2_FPEF category as an independent predictor of HF rehospitalization in the overall cohort (odds ratio: 3.4, CI: 2.4–4.9, *p* < 0.01) as well as in patients with and without overlapping conditions. Furthermore, a H_2_FPEF score ≥ 6 was independently associated with higher mortality rates (hazard ratio: 1.8, CI: 1.2–2.6, *p* < 0.01) in the Cox regression analysis. **Conclusions**: Ambulatory patients presenting for suspected CAD and meeting HFpEF screening criteria face elevated risks for rehospitalizations over six years. Regardless of concomitant diagnoses, quantifying cardiac damage with the H_2_FPEF score helps in risk-stratifying patients for HF hospitalization and mortality.

## 1. Introduction

Heart failure with preserved ejection fraction (HFpEF) has often been described as a new “epidemic” among cardiovascular diseases, accounting for up to 50% of the causes of heart failure (HF) hospitalizations [1]. People older than 75 years of age are those primarily affected, but disease prevalence is still high among people aged 60–75 years [2,3,4]. The future change in population demographics will thus increase the trajectory of this epidemic. Despite this worrisome trend, only limited therapeutic options exist to date [5,6].

HFpEF is a heterogeneous syndrome, and correct diagnosis is challenging in clinical practice as a high and variable comorbidity burden might obscure initial symptoms and modify the clinical trajectory [7]. Coronary artery disease (CAD) and atrial fibrillation (Afib) are among the most common comorbid cardiac conditions in HFpEF. CAD and Afib are present in at least two-thirds of patients hospitalized for HFpEF and have been shown to be associated with mortality in this group [8,9].

While the current guidelines suggest a practical screening algorithm, the current reference standard includes invasive detection of elevated filling pressures [10]. Over the last few years, specific diagnostic algorithms have been suggested in order to better classify and risk-stratify HFpEF patients non-invasively [11,12]. Amongst them, the H_2_FPEF score is the only evidence-based score, developed to predict the presence of invasively determined elevated filling pressures. These should help to identify patients early during the course of the disease in order to implement treatment measures. However, effective treatment is hampered by the heterogeneity of events that need to be prevented. Exemplarily, SGLT2 inhibition has been shown to reduce HF hospitalizations but not overall hospitalizations in an elderly HFpEF population and showed no reduction in mortality rates [6], and semaglutide shows promise in reducing symptoms in this population of patients [13]. Simple and easily assessable scores, like the H_2_FPEF score, and clinical categories have the largest potential to aid in determining the appropriate treatment paths and intensity, especially in patients undergoing screening for HF at the time when presenting at a cardiology clinic for various reasons [14].

We therefore aimed to investigate the clinical trajectory of patients undergoing cardiologic counseling for suspected CAD but fulfilling non-invasive HFpEF criteria and investigate whether screening by the H_2_FPEF score could help in decision-making in this group of patients.

## 2. Materials and Methods

### 2.1. Patients

Patients with suspected HFpEF were identified from the LIFE-Heart study, a large observational cohort study [15]. From the pool of patients who were referred to our tertiary center for invasive diagnostic procedures due to their high cardiovascular risk and a high pre-test probability for CAD, we selected patients who had a positive HFpEF screening with fulfilment of the European Society of Cardiology (ESC) 2016 guideline criteria [11]. Specifically, these were signs/symptoms compatible with HF, the presence of a left ventricular ejection fraction (LV-EF) ≥ 50%, elevated N-terminal prohormone of brain natriuretic peptide (NT-pro-BNP) ≥ 125 ng/L, and evidence of structural heart diseases defined by either diastolic dysfunction (E/E’ ≥ 13), left ventricular (LV) hypertrophy (♂: ≥115 g/m^2^, ♀: ≥95 g/m^2^), and/or left atrial (LA) dilatation (≥34 mL/m^2^ or anteroposterior diameter ≥ 23 mm/m^2^) [10]. Recruitment took place between January 2006 and December 2014 [15].

Exclusion criteria were the presence of an acute coronary syndrome according to the current definition [16], LV-EF < 50%, missing classification data, and inconclusive classification.

Patients were comprehensively phenotyped, involving medical questionnaires and examinations, electrocardiography, invasive coronary angiography, echocardiography, and laboratory testing, as detailed previously [15]. Severe diastolic dysfunction (restrictive pattern) was defined as an E/e’ > 14 and E/A > 2 in the case of sinus rhythm [17].

Medical therapy was given and up-titrated to the maximal tolerated doses according to the current guidelines of the European Society of Cardiology. LIFE-Heart meets the ethical standards of the Declaration of Helsinki, was approved by the Ethics Committee of the medical faculty of the University of Leipzig (Reg. No 276/05-ek, 1 November 2005), and is registered at ClinicalTrials.gov (No NCT00497887). Written informed consent was collected from all patients.

### 2.2. Patient Classification

The H_2_FPEF score [11] was calculated for all patients at presentation. Patients were subdivided into three categories (“low-risk”, “intermediate-risk”, and “high-risk”) on the basis of H_2_FPEF scores (0–1, 2–5, and ≥6, respectively). The presence of CAD was verified according to the invasive coronary status and was defined as the presence of any stenosis ≥50% as reported by the operator. Afib was defined as the presence of Afib on the resting 12-lead-electrocardiogram or the history of Afib according to the patient and hospital charts. Treatment strategies regarding CAD and Afib were at the discretion of the attending cardiologist.

The presence of treatable clinical conditions potentially causing or camouflaging HF symptoms was considered aggravating conditions and defined as “overlapping conditions” at presentation. These included the presence of tachy- or bradyarrhythmia at presentation, a chronic coronary syndrome with intervention, the presence of an intracardiac shunt, more-than-moderate valve disease, sick sinus syndrome, chronic obstructive pulmonary disease, anemia with hemoglobin <7 g/dL, and pulmonary embolism.

### 2.3. Follow-Up

Patients were systematically followed-up with to January 2021 for the occurrence of rehospitalization and reason for rehospitalization using the hospital’s integrated medical record system and inquiries at central residence registers. Regarding survival status, the last register enquiry was used for survival analyses (December 2020). Every hospitalization was reviewed, and the cause of hospitalization was determined. HF hospitalization for HFpEF was defined as hospitalization for dyspnea in the absence of the predominant treatment for one of the overlapping conditions as detailed above (i.e., the ICD-10 at patient’s discharge was HF).

### 2.4. Statistical Analysis

Normality of data was assessed using Shapiro–Wilk tests. Continuous variables are shown as mean ± standard deviation or median with interquartile range (IQR) in the case of normal and non-normal distributions, respectively. Categorial variables are shown as frequencies and percentages. Differences between groups were tested by means of Student’s *t*-tests or Mann–Whitney U tests; one-way ANOVAs or Kruskal–Wallis-K-Tests; as well as Chi-square tests (Pearson) where appropriate.

Logistic regression analyses were used to evaluate predictors for hospitalizations, with calculations of odds ratios (OR) with 95% confidence intervals (CIs) accordingly. Univariable and multivariable predictors of mortality were assessed using Cox regression analyses, with the calculation of hazard ratios (HRs) and respective CIs. Only univariable predictors with a *p* < 0.05 were considered for Cox multivariable analyses. The natural logarithm of NT-pro-BNP was calculated in order to normalize the data distribution. Kaplan–Meier estimates were used to display different survival rates among the analyzed subgroup. A *p*-value < 0.05 for the Log rank test was considered significant. Censored data were displayed as well.

Receiver operating characteristic (ROC) statistics were employed to evaluate the predictive performance of the linear H_2_FPEF score for heart failure (HF) hospitalization, as indicated by the area under the curve (AUC).

In general, a two-sided *p*-value < 0.05 was considered statistically significant for all applied tests. Statistical analyses were performed using IBM SPSS Statistical Software (version 25.0) and Jupyter Notebook using the data analysis packages numpy, pandas, matplotlib, lifelines, and seaborn in Python 3.37.

## 3. Results

### 3.1. Patient Population and Definition of Cohorts

**Figure 1:**
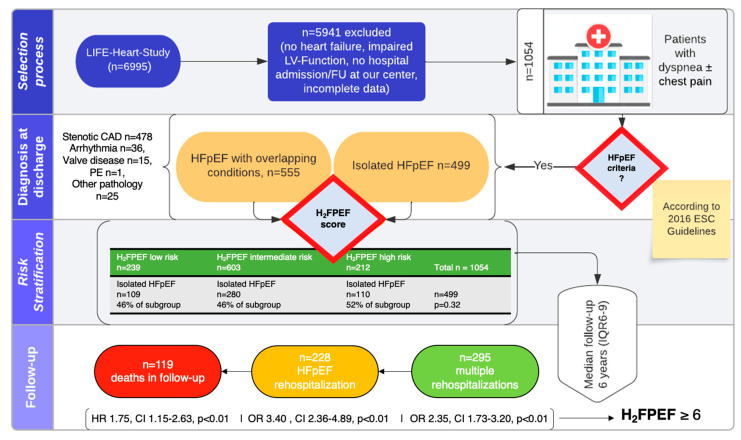
Flowchart with study design and follow-up. Legend: CAD = coronary artery disease; ESC = European Society of Cardiology; FU = follow-up, HFpEF = heart failure with preserved ejection fraction; H_2_FPEF = scoring system based on age, BMI, atrial fibrillation, pulmonary hypertension, elevated filling pressures, and arterial hypertension; HR = hazard ratio; IQR = interquartile range; OR = odds ratio; PE = pulmonary embolism; LV-Function = left ventricular systolic function; valve disease = moderate or severe valve disease.

Within the observational period, 1054 patients from the LIFE-Heart study, presenting at the Heart Center Leipzig at University of Leipzig for suspected CAD, fulfilled the inclusion criteria, constituting the overall cohort. The study flow is depicted in Figure 1.

The baseline characteristics are shown in Table 1. The overall cohort had a mean age of 66 ± 10 years, and patients were predominantly male (60%). All of the patients reported exertional dyspnea, predominantly in New York Heart Association (NYHA) class II, and 42% presented with chest pain at presentation. The cohort is characterized by a marked cardiovascular risk profile, namely, consisting of obesity, arterial hypertension, diabetes, history of smoking, and a history of previous coronary interventions. The majority of patients showed preserved renal function with only 4% presenting with chronic kidney diseases ≥ stage 4 [18]. On average, NT-pro-BNP levels, peripheral inflammatory markers (C-reactive protein, CRP, and interleukin-6, IL 6), and high-sensitivity (hs)-troponin-T were moderately increased compared to normal values. Patients demonstrated a normal LV-EF, evidence of elevated LV filling pressures, and moderate LA dilatation, with preserved LV dimensions. LA dilatation was present in 63%, LV hypertrophy in 86%, and severe diastolic dysfunction in 24% of cases. A moderate valvular lesion (mitral regurgitation and aortic stenosis) was present in 11% of patients. Afib was present in 26% of patients with 38% having paroxysmal Afib, 39% experiencing persistent Afib, and 22% having permanent Afib within the subgroup.

When applying the H_2_FPEF score to the overall cohort, 239 patients (22.6%) were classified as low-risk, 603 patients (57.2%) as intermediate-risk, and 212 patients (20.1%) as high-risk for HFpEF. Differences in the baseline characteristics between these groups are reported in Table 1. A higher risk category was associated with older age, more frequent chest pain, a more pronounced cardiovascular risk profile, worse renal function, higher NT-pro-BNP and hs-troponin-T, as well as CRP elevation. The proportion of patients with higher NYHA classes gradually increased with higher-risk H_2_FPEF categories (*p* < 0.01). The higher-risk patients had evidence of smaller LV cavities and estimates of higher LV filling pressures, more LA dilatation, and higher right ventricular systolic pressures but similar incidence of valvular disease. At inclusion, there was no significant difference in the incidence of overlapping pathology (54.4%, 53.6%, and 48.1% across the risk stages, *p* = 0.32). High-risk patients exhibited the lowest frequencies of invasively diagnosed CAD but the highest frequencies of Afib (see Table 1).

At presentation, 47% of patients (*n* = 499) exhibited symptoms exclusively attributable to HFpEF, thus termed the “isolated HFpEF group”. Their baseline characteristics are detailed in Supplementary Table S1, along with subgroup variations based on the H_2_FPEF score at presentation. Significant differences in demographic, laboratory, and echocardiographic parameters persisted across the H_2_FPEF categories in this cohort when compared to the overall cohort (refer to Supplementary Table S1).

The subgroup of “isolated HFpEF” at presentation shared common features to the subgroup of “HFpEF with overlapping conditions” and, therefore, the overall cohort when considering laboratory and echocardiographic parameters (see Supplementary Tables S1 and S2). However, it exhibited a greater representation of female patients when compared to the overall cohort (49% vs. 31%, *p* < 0.05) and revealed a higher prevalence of Afib (29% vs. 23%, *p* < 0.02). Only 7% of patients in the overall cohort and 6.6% in the cohort of patients with “isolated HFpEF” presented overt signs and symptoms of cardiac decompensation at presentation (*p* = 0.21).

In the invasive coronary angiography, 49.6% of patients in the overall cohort had evidence of CAD with indication for intervention (*n* = 4 with conservative treatment, i.e., optimal medical therapy). The rate of having a previous history of CAD was significantly lower among patients with isolated HFpEF at inclusion if compared to that of the subgroup of HFpEF with overlapping conditions (3.6% vs. 27%, *p* < 0.01).

### 3.2. Rehospitalizations

**Figure 2:**
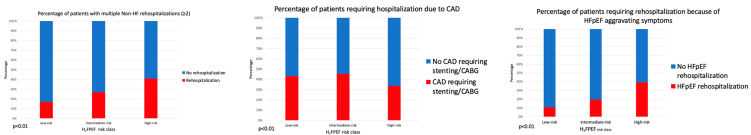
Overall rehospitalizations according to H_2_FPEF category (upper), percentage of patients with a HFpEF-related rehospitalization (middle), and percentage of coronary hospitalizations (lower left corner). CAD = coronary artery disease; CABG = coronary artery bypass graft; HFpEF = heart failure with preserved ejection fraction; H_2_FPEF = scoring system based on age, BMI, atrial fibrillation, pulmonary hypertension, elevated filling pressures, and arterial hypertension. Risk classes are defined as low-risk for 0–1, intermediate-risk for 2–5, and high-risk for ≥6, respectively.

Over a median follow-up period of 6 years (IQR 6–8), 569 patients (54%) encountered rehospitalization, with 295 patients (28%) experiencing more than one recurrent hospitalization. The average number of rehospitalizations significantly increased across the H_2_FPEF-score risk classes (*p* < 0.01, see Table 1 and Figure 2).

In total, 228 patients (22%) experienced an HF rehospitalization during the follow-up period. Among these, 88 patients were from the isolated HFpEF group (17.6% of the subgroup), while 140 were from the group of patients presenting with overlapping conditions at inclusion (25% of the subgroup, *p* < 0.01 between the subgroups). The risk of hospitalization due to HF during the follow-up significantly increased across the H_2_FPEF risk classes, both in the overall cohort and in the subgroup with isolated HFpEF at presentation.

The main components of non-HF hospitalizations at first rehospitalization according to the ICD-10 at discharge in the overall cohort were elective Afib ablation (*n* = 20, 3.5%), valve disease (*n* = 42, 7.3%), CAD or suspicion of CAD (*n* = 274, 48.2%), pulmonary embolism (*n* = 2, 0.3%), syncope (*n* = 22, 3.8%), arrhythmia (i.e., symptomatic tachy- or bradyarrhythmia, *n* = 32, 5.6%), and other (*n* = 47, 8.3%), as detailed in Supplementary Figure S2.

At first rehospitalization, 73 patients in the initial isolated HFpEF group (14.6%) and 84 patients in the group of HFpEF with overlapping conditions (15.1%) experienced a rehospitalization due to significant CAD which required revascularization (*p* = 0.81 between the subgroups). The proportion of patients with CAD rehospitalization was significantly lower in patients with a H_2_FPEF score ≥ 6 (Figure 2).

In total, 69 patients (6.5% of the overall cohort) were hospitalized for severe valvular disease which required intervention (subgroup difference between isolated HFpEF and HFpEF with overlapping conditions: 3.4% vs. 9.3%, *p* < 0.01).

### 3.3. Predictors of Rehospitalization

A diagnosis of CAD at inclusion was a significant predictor of overall rehospitalization (OR: 5.66, CI: 3.76–8.50, *p* < 0.01) but was inversely related to the risk of HF rehospitalizations (OR: 0.50, CI 95%: 0.34–0.73).

A history of Afib at inclusion predicted the risk of overall rehospitalizations (OR: 1.21, CI: 1.13–1.31, *p* < 0.01), and Afib was predictive for HF rehospitalization (OR: 2.56, CI: 1.92–3.44, *p* < 0.01). HF rehospitalization rates among the types of Afib were 52%, 66%, and 60%, for paroxysmal, persistent, and permanent Afib, respectively (*p* = 0.29).

Similarly, assignment to the high-risk H_2_FPEF category at inclusion was associated with overall rehospitalizations (OR: 2.17, CI: 1.58–2.98, *p* < 0.01), and on multivariable logistic regression, a high-risk H_2_FPEF category at inclusion emerged as a single independent predictor of HFpEF rehospitalization (OR: 3.40, CI 95%: 2.36–4.89, *p* < 0.01).

This was true in the overall cohort (Table 2) as well as among the subgroups of “isolated HFpEF” and “HFpEF with overlapping conditions” (Supplementary Tables S3 and S4). When considered linearly, the H_2_FPEF score showed moderate predictive value for HF hospitalizations on ROC in the overall cohort (AUC: 0.63, CI: 0.59–0.66, *p* < 0.01), in patients with CAD (AUC: 0.60, CI: 0.56–0.65, *p* < 0.01), as well as in patients without CAD (AUC: 0.65, CI: 0.59–0.70, *p* < 0.01) (Supplementary Figure S3).

### 3.4. Mortality

**Figure 3:**
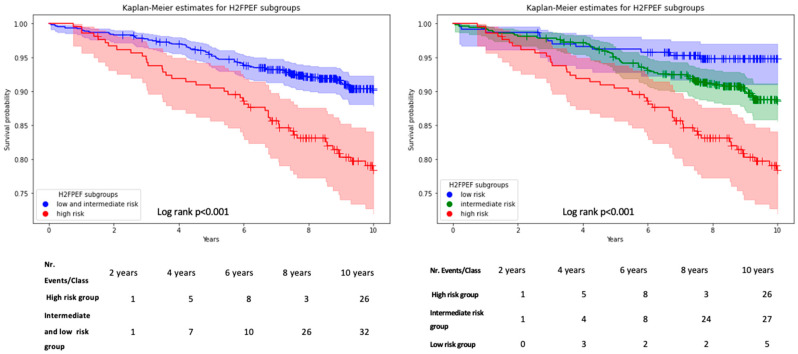
Kaplan–Meier estimates for patients according to H_2_FPEF category. Ticks represent censored data. Legend: H_2_FPEF = scoring system based on age, BMI, atrial fibrillation, pulmonary hypertension, elevated filling pressures, and arterial hypertension. Risk classes are defined as low risk for 0–1, intermediate or middle risk for 2–5, and high-risk for ≥6, respectively.

Overall, 119 patients (11.3%) died during the follow-up period. Probability of survival decreased with higher H_2_FPEF category at inclusion (low: *n* = 12, 5%; intermediate: *n* = 64, 10.6%; high: *n* = 43, 20.3% *p* < 0.01) and survival time decreased (log-rank *p* < 0.01, Figure 3). In the subgroup of isolated HFpEF *n* = 57 (11.4%) patients died during follow-up as opposed to *n* = 62 (11.1%) in patients with overlapping symptoms (*p* = 0.89). Similarly, survival times in the high-risk group did not differ between patients with and without overlapping syndromes (Supplementary Figure S4).

In the Cox regression analyses, the univariable predictors for the occurrence of all-cause mortality were age (HR: 1.10, CI: 1.08–1.13, *p* < 0.01), male sex (HR: 1.88, CI: 1.25–2.82, *p* < 0.01), Afib (HR: 1.95, CI: 1.35–2.82, *p* < 0.05), H_2_FPEF high-risk class (HR: 2.77, CI: 1.89–4.07, *p* < 0.01), diabetes mellitus (HR: 1.63, CI: 1.14–42.34, *p* < 0.01), a higher E/e’ ratio (HR: 1.09, CI: 1.0.4–1.14, *p* < 0.01), a higher NYHA class at presentation (HR: 2.72, CI: 1.91–3.86, *p* < 0.01), and a higher lnNT-pro-BNP (HR: 3.86, CI: 2.58–5.78, *p* < 0.01).

Independent significant predictors in the stepwise multivariable analysis (backward selection), excluding variables found in the H_2_FPEF score in order to avoid co-linearity (i.e., age, presence of Afib, body mass index, and E/e’), were male sex (HR: 1.73, CI: 1.15–2.60, *p* < 0.01), H_2_FPEF high-risk category (HR: 1.75, CI: 1.15–2.63, *p* < 0.01), and lnNT-pro-BNP (HR: 1.57, CI: 1.19–2.63, *p* < 0.01). All details are summarized in Table 3.

## 4. Discussion

Here we present the clinical follow-up of a large real-world cohort of patients at elevated cardiovascular risk who fulfilled HFpEF criteria while being evaluated for the suspicion of CAD. The key findings of this study can be summarized as follows:

1. Patients presenting with a positive screening for HFpEF while being evaluated for CAD exhibit an important phenotypic heterogeneity with overlapping comorbidities in 53% of patients, and only 20% are classified as high-probability HFpEF based on the H_2_FPEF score.
2. Rehospitalizations were common, but reasons for rehospitalization varied. The H_2_FPEF score but not the presence of overlapping comorbidities was strongly associated with HF-specific rehospitalizations.
3. The H_2_FPEF score is a potent predictor of mortality in this heterogeneous patient cohort.

HFpEF is an underestimated syndrome with increasing prevalence and high morbidity and mortality. The ‘HFpEF’ epidemic has been attributed to the aging societies and to a multitude of cardiovascular risk factors, generating a low-grade systemic inflammation, vascular dysfunction, and the HFpEF phenotype [5,19]. Within this phenotype, considerable heterogeneity exists, and overlapping comorbidities may mimic or conceal the presence of chronic HF. To positively influence outcomes in HFpEF patients, it is crucial to understand the mechanisms of disease trajectory, including reasons for rehospitalization and mortality. This is illustrated by the fact that SGLT2 inhibition has recently been shown to reduce cardiovascular rehospitalizations but not overall rehospitalizations in HFpEF.

Given the complexity of the syndrome with the recommendations for sophisticated workups in specialized centers [12] and the scope of the problem in everyday practice, it is desirable to have clinical tools easily and readily available to guide decision-making to further refer patients to specialized HF clinics.

Our study investigated patients at elevated cardiovascular risk undergoing workups for suspicion of CAD, fulfilling the non-invasive screening criteria for HFpEF according to the 2016 ESC guidelines, which is currently used in our practice as a screening tool. As suspected, we observed a phenotypic heterogeneity with 52.7% of patients with overlapping syndromes at presentation. In comparison to other HFpEF cohorts like the “Olmsted County” cohort (*n* = 2762) from Gerber et al. [20], the “FHS” cohort (*n* = 1038) from Lam et al. [21], and the “EFFECT-Study” from Bhatia et al. [22], our patients were, on average, 10 years younger and with male prevalence (60% in the overall cohort and 51% in the isolated HFpEF cohort versus 35–42% in the aforementioned studies).

A possible explanation for these discrepancies is the differences in inclusion criteria. For the “Olmsted County” cohort and the “FHS” cohort, the Framingham criteria with the documentation of preserved LV-EF were applied, whereas in the “EFFECT-Study”, the ICD-Code at discharge and the LV-EF control within 90 days of discharge were considered for HFpEF diagnosis. Our cohort was made up of patients undergoing workups for stable CAD, which is known to show a male predominance [23]. However, overall, only half of our patients exhibited significant CAD, with comparable revascularization rates to those of other studies [20,21,22], further supporting our approach to consider a more general HFpEF phenotype in order to evaluate the following clinical trajectory.

One quarter of the patients had Afib, which was consistent with other cohorts [22]. Although all patients fulfilled the ESC guideline criteria, when further stratifying the cohort according to H_2_FPEF categories, we found a high likelihood of HFpEF in about 20% of patients, a low likelihood in 23% of patients, and an intermediate HFpEF probability in the majority of patients. This observation was true-independent of the presence of overlapping comorbidities and consistent with observations in other cohorts [24].

Comorbidities (obesity, renal dysfunction, and diabetes) and cardiac abnormalities (diastolic dysfunction, LA-dilatation, and right ventricular pressures) commonly associated with HFpEF gradually increased across the H_2_FPEF risk groups. Notably, in the high-risk category, the presence of CAD was inversely correlated with HF hospitalizations. This is partly explained by the rigorous way to define HF hospitalizations in our study, making HF hospitalization counts in patients with CAD less likely. In clinical practice, a clear cut between HF and CAD hospitalization might be less obvious in some cases. However, CAD is a very common comorbidity in HFpEF, and effective therapies for symptom alleviation and secondary prevention exist [20,25,26]. Surprisingly, in this cohort undergoing cardiologic workups for CAD, evidence of the latter was not predictive of mortality. In contrast, markers associated with a more advanced global cardiomyopathy or cardiac damage were related to both HF hospitalizations and mortality [27]. This included a history of Afib (independent of paroxysmal, persistent or permanent) and, importantly, a high H_2_FPEF score.

The H_2_FPEF score entails six different demographic and functional patient characteristics, which, when combined with a weighted score, have been shown to inform on the probability of a HFpEF diagnosis derived from the reference standard of dynamic invasive exercise testing [11]. Notably, this score includes hemodynamic and demographic parameters that reflect both advanced cardiomyopathy and baseline risk. Consequently, a higher H_2_FPEF score has been shown to be associated with more advanced cardiac injury and adverse prognosis in terms of cardiovascular events and HF rehospitalizations in patients with HFpEF and stable outpatients with cardiovascular risk factors [28,29].

We opted to utilize the H_2_FPEF categories for HFpEF probabilities as risk stratifiers, facilitating intuitive simultaneous diagnostic and prognostic considerations.

The fact that this score rather than overlapping comorbidities was the most potent predictor of HF hospitalizations and mortality in our cohort underscores the important role of more diffuse advanced cardiac damage in our everyday patients, which is not necessarily present with specific signs of HF decompensation. This simple point-score based screening identifies patients who would most likely benefit from specific medical therapies designed to avoid HF deterioration and HF hospitalization and might help to allocate specialized treatment pathways to these patients. In addition, the application of this score could not only improve the risk–benefit ratios and numbers needed to treat for specific therapies but can also inform strategies for risk enrichment in dedicated trials.

### Strengths and Limitations

Although we present a fairly large real-world patient sample of patients with suspected HFpEF, this study has some limitations. Given the cohort of patients being referred to workups for suspected CAD, which were predominantly male and relatively young, the findings might not be generalizable to more general HFpEF cohorts. However, the clinical scenario illustrated in this study is very common, and the suggested approach helps to further prioritize referrals to HF clinics. Detailed insights into individual medical therapies throughout the duration of the study are not available. However, considering that patients were treated at a single tertiary care center according to best medical practice, we contend that this does not diminish the validity of our observations regarding risk stratification by the H_2_FPEF score in these patients.

Although we thoroughly investigated reasons for hospitalizations, scrutinizing specific clinical trajectories, the mode of death was not ascertained. Instead, all-cause mortality was assessed since the cause of death could not be identified in public registries. The generalizability of the results needs substantiation in broader demographics. Further research in diverse patient populations, and including the assessment of cardiovascular mortality, may strengthen its validity and applicability.

## 5. Conclusions

In summary, these data emphasize the heterogeneity of general cardiovascular patients with a positive HFpEF screening and the challenges in accurately predicting the clinical trajectories. The H_2_FPEF score enabled the quantification of more advanced overall cardiac damages independent of overlapping comorbidities. While rehospitalizations were common, the admission reasons varied. A high H_2_FPEF score was the strongest independent predictor of HF hospitalizations and mortality and should be used to guide referrals to specific HF clinics in clinical practice and risk enrichment in clinical trials. 

## Figures and Tables

**Table 1:** Baseline characteristics and stratification according to H_2_FPEF score in overall cohort.

Variables	All Patients *n* = 1054	H_2_FPEF Low-Risk *n* = 239	H_2_FPEF Intermediate-Risk *n* = 603	H_2_FPEF High-Risk *n* = 212	*p*-Value
**Patient characteristics**					
Age, years	66 ± 10	58 ± 10	68 ± 9	70 ± 7	<0.01
Female sex, *n*. (%)	421 (40%)	84 (35%)	256 (42%)	81 (38%)	0.13
NYHA class, *n*. (%)	NYHA II: 977 (93%) NYHA III: 68 (6.5%) NYHA IV: 9 (0.5%)	NYHA II: 233 (97%) NYHA III: 3 (1.5%) NYHA IV: 3 (1.5%)	NYHA II: 568 (94%) NYHA III: 31 (5%) NYHA IV: 4 (1%)	NYHA II: 176 (83%) NYHA III: 34 (16%) NYHA IV: 2 (1%)	<0.01
Chest pain, *n*. (%)	445 (42%)	152 (64%)	212 (35%)	81 (38%)	<0.01
BMI, kg/m^2^	30 ± 5	28 ± 4	31 ± 5	31 ± 5	<0.01
Obesity, *n*. (%)	487 (46%)	35 (15%)	324 (54%)	128 (60%)	<0.01
Diabetes, *n*. (%)	371 (35%)	43 (18%)	217 (36%)	111 (52%)	<0.01
Arterial hypertension, *n*. (%)	656 (62%)	139 (58%)	384 (64%)	133 (63%)	0.33
Smoking, *n*. (%)	368 (35%)	84 (35%)	207 (34%)	77 (36%)	0.87
Hx CAD intervention, *n*. (%)	169 (16%)	26 (11%)	107 (18%)	36 (17%)	0.05
Dx of CAD, *n*. (%)	523 (50%)	125 (52%)	311 (52%)	87 (41%)	0.02
Atrial fibrillation, *n*. (%)	279 (26%)	0 (0%)	67 (11%)	212 (100%)	<0.01
HF hospitalization, *n*. (%)	499 (47%)	109 (46%)	280 (46%)	110 (52%)	0.33
Non-HF hospitalization, *n*. (%)	555 (53%)	130 (54%)	323 (54%)	102 (48%)	0.33
**Laboratory Values**					
eGFR, mL/min/1.73 m^2^	66 ± 25	76 ± 27	63 ± 24	63 ± 23	<0.01
eGFR < 30, *n*. (%)	51 (5%)	5 (2%)	33 (5%)	13 (6%)	0.07
NT-proBNP, ng/L	540 (178–543)	217 (164–341)	280 (177–490)	413 (242–1112)	0.05
CRP, mg/L	5.0 ± 9.8	4.5 ± 10.8	4.8 ± 9.3	6.0 ± 10.1	0.02
IL-6, pg/mL	5.18 ± 12.75	5.74 ± 23.93	4.96 ± 6.98	5.16 ± 4.99	0.80
Troponin T, pg/mL	9.1 (6.0–12.9)	7.8 (5.1–10.8)	10.7 (7.0–11.6)	10.8 (8.7–14.3)	<0.01
**Echocardiographic Parameters**					
LV-EF, %	61 ± 7	61 ± 6	61 ± 7	62 ± 7	0.21
E/e’	10.4 ± 3.9	8.9 ± 3.0	10.6 ± 3.8	12.8 ± 4.6	<0.01
LV-EDV index, mL/m^2^	53 ± 18	55 ± 18	52 ± 17	49 ± 18	0.01
LV-Mass index, g/m^2^	138 ± 40	134 ± 37	141 ± 42	140 ± 42	0.03
LA diameter index, mm/m^2^	24 ± 4	23 ± 3	24 ± 3	26 ± 4	<0.01
TAPSE, mm	21 ± 4	21 ± 4	21 ± 4	20 ± 4	0.53
TR Vmax, m/s	2.5 ± 0.8	2.5 ± 1.0	2.4 ± 0.5	2.8 ± 0.6	<0.01
Moderate valvular disease, *n*. (%)	120 (11%)	13 (5%)	64 (11%)	43 (20%)	<0.01
**Events during follow-up**					
Follow-up time, years	6 (IQR 6–9)	6 (IQR 6–9)	6 (IQR 6–8)	6 (IQR 6–8)	0.72
HF rehospitalization, *n*. (%)	228 (22%)	26 (11%)	119 (20%)	83 (39%)	<0.01
Average number of rehospitalizations, *n*.	1.15 ± 1.7	0.75 ± 1.4	1.13 ± 1.7	1.77 ± 1.9	<0.01
All-cause mortality, *n*. (%)	119 (11%)	12 (5%)	64 (11%)	43 (20%)	<0.01

For continuous parameters, we present mean and SD. *p*-values refer to a global test of the three H_2_FPEF risk groups. NYHA Class = New York Heart Association Class (1–4), BMI = body mass index, CAD = coronary artery disease, Dx = diagnosis, HF = heart failure, Hx = history, eGFR = estimated glomerular filtration rate, LV-EF = left ventricular ejection fraction, LV-EDV = left ventricular end-diastolic volume, LA = left atrial, TR Vmax = peak velocity of tricuspid valve regurgitation in CW doppler, TAPSE = tricuspid annular plane systolic excursion. Percentages equal to or greater than 0.5 were rounded to the larger integer.

**Table 2:** Logistic regression for HF hospitalization in overall cohort.

	Logistic Regression Model (Univariate)				Logistic Regression Model (Multivariable)			
	95.0% CI for EXP(B)				95.0% CI for EXP(B)			
	EXP(B)	Lower	Upper	*p*-Value	EXP(B)	Lower	Upper	*p*-Value
Male sex	1.21	0.89	1.83	0.21	1.20	0.87	1.64	0.26
lnNTproBNP	1.04	0.87	1.24	0.64	1.00	0.78	1.27	0.99
NYHA-class	0.73	0.42	1.26	0.26	0.61	0.28	1.31	0.20
H_2_FPEF high-risk	3.09	2.23	4.29	<0.01	3.40	2.36	4.89	<0.01

H_2_FPEF high risk = H_2_FPEF score equal to or greater than 6 as binary variable, lnNT-pro-BNP = natural logarithm of the NT-pro-BNP at baseline, NYHA-Class = New York Heart association class (1–4), EXP(B) is considered equivalent to odds ratio. Hosmer and Lemeshow test *p* = 0.28, Cox and Snell R Square *p* = 0.5, Nagelkerke R Square *p* = 0.8 for multivariable model, age, Afib, BMI, and E/e’ were not inputted due to significant co-linearity with H_2_FPEF score.

**Table 3:** Univariable and multivariable Cox-proportional models for all-cause mortality. Multivariable model was realized inputting significant variables from the univariate ones, excluding those included in an integrated score.

	Cox-Proportional Model (Univariate)				Cox-Proportional Model (Multivariable)			
		95.0% CI for HR				95.0% CI for HR		
	HR	Lower	Upper	*p*-Value	HR	Lower	Upper	*p*-Value
Age (years)	1.10	1.08	1.13	<0.01	-	-	-	-
Male sex	1.88	1.25	2.82	<0.01	1.73	1.15	2.60	<0.01
CAD	0.80	0.47	1.37	0.41	-	-	-	-
Afib	1.95	1.35	2.82	<0.01	-	-	-	-
H_2_FPEF High-risk	2.78	1.89	4.07	<0.01	1.75	1.15	2.63	<0.01
lnNT-proBNP	3.86	2.58	5.78	<0.01	1.57	1.19	2.60	<0.01
NYHA-class	2.72	1.91	3.86	<0.01	-	-	-	n.s.
Diabetes mellitus type 2	1.63	1.14	2.34	0.01	-	-	-	n.s.
E/e‘ average	1.09	1.05	1.14	<0.01	-	-	-	-

CAD = coronary artery disease, Afib = atrial fibrillation, E/e’ average = echocardiographic parameter for the measurement of diastolic function, H_2_FPEF high risk = H_2_FPEF score equal or greater than 6 as binary variable, lnNT-pro-BNP = natural logarithm of the NT-pro-BNP at baseline, NYHA-Class = New York Heart Association class (1–4), n.s. = not significant.

## Data Availability

No new data were created or analyzed in this study. Data sharing is not applicable to this article.

## Supplementary Materials

The following supporting information can be downloaded at: https://www.mdpi.com/article/10.3390/jcm13072092/s1, Table S1. Baseline characteristics of patients with isolated HFpEF and stratification according to H_2_FPEF Score in the cohort of isolated HFpEF; Table S2. Baseline characteristics of patients with isolated HFpEF and stratification according to H_2_FPEF Score in the cohort of HFpEF with overlapping conditions; Table S3. Logistic Regression for HF-hospitalization in cohort of patients with HFpEF and overlapping conditions at presentation; Table S4. Logistic Regression for HF-hospitalization in cohort of patients with isolated HFpEF; Figure S1. Classification of the overall cohort with detailed reason of hospitalization for the subgroup of HFpEF with overlapping conditions; Figure S2. Detailed reasons of first rehospitalization; Figure S3. ROC curves for HFpEF hospitalization and death in overall cohort, CAD subgroup and Non-CAD subgroup; Figure S4. KM-estimates for isolated HFpEF and HFpEF with overlapping conditions in H_2_FpEF score high risk group.

## Abbreviations

Afib	atrial fibrillation
ANOVA	analysis of variance
CAD	coronary artery disease
CI	confidence interval
COPD	chronic obstructive pulmonary disease
CRP	C-reactive protein
ECG	electrocardiogram
EDV	end-diastolic volume
eGFR	estimated glomerular filtration rate
ESC	European Society of Cardiology
HF	heart failure
HFpEF	heart failure with preserved ejection fraction
HR	hazard ratio
Hs-troponin	high-sensitivity troponin
IQR	interquartile range
ICD-10	International Statistical Classification of Diseases and Related Health Problems (WHO version 10)
LA	left atrium
LV	left ventricle
LV-EF	left ventricular systolic ejection fraction
NYHA	New York Heart Association
NTproBNP	N-terminal-pro hormone B-type natriuretic peptide
OR	odds ratio
PVI	pulmonary vein ablation
