# Supplementary material for: Clinical Trajectory and Risk Stratification for Heart Failure with Preserved Ejection Fraction in a Real-World Cohort of Patients with Suspected Coronary Artery Disease
Source: J Clin Med. 2024 Apr 3;13(7):2092. doi: 10.3390/jcm13072092 (PMC11012700; doi:10.3390/jcm13072092)
Supplement: Supplementary file 1 [file jcm-13-02092-s001.zip › jcm-2883506-supplementary.pdf]

Supplementary Material

Clinical trajectory and risk-stratification for heart failure with preserved ejection fraction in a real-world cohort of patients with suspected coronary artery disease

Guglielmo Gioia, MD[1]; Karl-Patrik Kresoja, MD[1]; Sebastian Rosch, MD[1]; Anne Schöber, MD[1]; Elias Harnisch, BSc[1]; Maximilian von Roeder, MD[1]; Markus Scholz, MD[2,3]; Sylvia Henger, MSc[3]; Berend Isermann, MD[4]; Holger Thiele, MD[1]; Philipp Lurz, MD, PhD[5]; Karl-Philipp Rommel, MD[1,6]

[1]Dept. of Cardiology, Heart Center at University Leipzig and Leipzig Heart Science, Leipzig, Germany
[2]LIFE – Leipzig Research Center for Civilization Diseases, University of Leipzig, Germany.
[3] Institute of Medical Informatics, Statistic and Epidemiology, University of Leipzig, Germany.
[4]Institute of Laboratory Medicine, Clinical Chemistry and Molecular Diagnostics, University Hospital Leipzig, Germany
[5]Dept. Of Cardiology, University Hospital Mainz, Mainz, Germany
[6]Cardiovacular Research Foundation, New York, New York, USA

Correspondence address:
Karl-Philipp Rommel, MD
Department of Internal Medicine/Cardiology
Heart Center Leipzig at University of Leipzig
Strümpellstraße 39, 04289 Leipzig, GERMANY
Phone: +49141865252583
karl-philip.rommel@medizin.uni-leipzig.de

Supplementary Tables / Figures

Table S1. *Baseline characteristics of patients with isolated HFpEF and stratification according to H_2FPEF Score in the cohort of isolated HFpEF.*

**Variables**	**All Patients with isolated HFpEF** n=499	**H_2FPEF Low-Risk** n=109	**H_2FPEF Intermediate-Risk** n=280	**H_2FPEF High-Risk** n=110	**p-value**
**Patient Characteristics**					
Age, years	65 ± 10	58 ± 10	67 ± 9	69 ± 7	<0.01
Female sex, n. (%)	246 (49%)	54 (50%)	148 (53%)	44 (40%)	0.07
NYHA class, n. (%)	NYHA II: 466 (93.4%)<br>NYHA III: 32 (6.4%)<br>NYHA IV: 1 (0.2%)	NYHA II: 109 (100%)<br>NYHA III: 0 (0%)<br>NYHA IV: 0 (0%)	NYHA II: 267 (95.4%)<br>NYHA III: 12 (4.2%)<br>NYHA IV: 1 (0.4%)	NYHA II: 90 (81.8%)<br>NYHA III: 20 (18.2%)<br>NYHA IV: 0 (0%)	<0.01
Chest pain, n. (%)	207 (41%)	62 (57%)	108 (39%)	37 (34%)	<0.01
BMI, kg/m²	31 ± 5	28 ± 4	32 ± 5	32 ± 5	<0.01
Obesity, n. (%)	254 (51%)	19 (17%)	165 (59%)	70 (64%)	<0.01
Diabetes, n. (%)	182 (36%)	19 (17%)	105 (38%)	58 (53%)	<0.01
Arterial hypertension, n. (%)	302 (61%)	61 (56%)	177 (63%)	64 (58%)	0.36
Smoking, n. (%)	106 (21%)	22 (20%)	50 (18%)	34 (31%)	0.87
Hx CAD intervention, n. (%)	18 (4%)	2 (2%)	11 (4%)	5 (5%)	0.51
Atrial fibrillation, n. (%)	148 (30%)	0 (0%)	38 (14%)	110 (100%)	<0.01
**Laboratory Values**					
eGFR, ml/min/1.73m²	68 ± 25	74 ± 24	66 ± 25	65 ± 24	<0.01
eGFR <30, n. (%)	19 (4%)	0 (0%)	11 (4%)	8 (7%)	0.02
NT-proBNP, ng/l	262 (172 - 510)	200 (155 - 323)	251 (177 - 490)	480 (230 - 1276)	<0.01
CRP, mg/l	2.3 (1.2-5)	1.8 (1-4)	2.5 (1.3-5)	2.8 (1.4-6.3)	<0.01
Troponin T, pg/ml	10.3 (6 - 12)	7.5 (4.5 - 10.8)	10.5 (6.0 - 11.8)	10.8 (8.6 - 14.4)	<0.01
**Echocardiographic Parameters**					
LV-EF, %	61 ± 7	60 ± 6	62 ± 6	61 ± 7	0.21
E/e'	10.5 ± 3.7	8 ± 2.7	10.75 ± 3.3	12.3 ± 3.3	<0.01
LV-EDV index, ml/m²	51 ± 18	54 ± 18	50 ± 17	49 ± 18	0.05
LV-Mass index, g/m²	138.5 ± 40	137 ± 23	138 ± 40	140 ± 39	0.86
LA diameter index, mm/m²	24 ± 3.7	23.72 ± 3.7	24 ± 3.5	26 ± 4	<0.01
TR Vmax, m/s	2.5 ± 0.7	2.32 ± 0.4	2.52 ± 0.9	2.57 ± 0.4	0.02
Moderate valvular disease, n. (%)	45 (9%)	4 (4%)	23 (8%)	18 (16%)	<0.01
**Events during follow-up**					
HF rehospitalization, n. (%)	88 (18%)	8 (7%)	41 (15%)	39 (35%)	<0.01
Average number of rehospitalizations, n.	0.46 ± 1.2	0.2 ± 0.5	0.4 ± 1.17	0.9 ± 1.4	<0.01
All-cause mortality, n. (%)	57 (11%)	4 (4%)	31 (11%)	22 (20%)	<0.01

NYHA Class = New York Heart Association Class (1-4), BMI = body mass index, CAD = coronary artery disease, HF = heart failure, Hx = history, FU = follow-up, eGFR = estimated glomerular filtration rate, LV-EF = left ventricular ejection fraction, LV-EDV = left ventricular enddiastolic volume, LA = left atrial, TR Vmax = peak velocity of tricuspid valve regurgitation in CW Doppler, TAPSE = tricuspid annular plane systolic excursion. Percentages equal or greater 0.5 were rounded to the larger integer.

Table S2. *Baseline characteristics of patients with isolated HFpEF and stratification according to H_2FPEF Score in the cohort of HFpEF with overlapping conditions.*

**Variables**	**All Patients HFpEF with overlapping conditions** n=555	**H_2FPEF Low-Risk** n=130	**H_2FPEF Intermediate-Risk** n=323	**H_2FPEF High-Risk** n=102	**P-value**
**Patient Characteristics**					
Age, years	67 ± 10	58.7 ± 11	68 ± 6	71 ± 4	<0.01
Female sex, n. (%)	175 (32%)	30 (23%)	108 (33%)	37 (36%)	0.05
NYHA class, n. (%)	NYHA II: 511 (92%)<br>NYHA III: 36 (6%)<br>NYHA IV: 8 (2%)	NYHA II: 124 (95%)<br>NYHA III: 3 (2.5%)<br>NYHA IV: 3 (2.5%)	NYHA II: 301 (93%)<br>NYHA III: 19 (6%)<br>NYHA IV: 3 (1%)	NYHA II: 86 (84%)<br>NYHA III: 14 (14%)<br>NYHA IV: 2 (2%)	<0.01
Chest pain, n. (%)	238 (43%)	90 (69%)	104 (32%)	44 (43%)	<0.01
BMI, kg/m²	30 ± 5	27.5 ± 3.9	30.1 ± 4.6	30.7 ± 4.8	<0.01
Obesity, n. (%)	233 (42%)	16 (12%)	159 (49%)	58 (57%)	<0.01
Diabetes, n. (%)	189 (34%)	24 (18%)	112 (35%)	53 (52%)	<0.01
Arterial hypertension, n. (%)	354 (64%)	78 (60%)	207 (64%)	69 (68%)	0.48
Smoking, n. (%)	262 (47%)	62 (48%)	157 (49%)	43 (42%)	0.52
Hx CAD intervention, n. (%)	151 (27%)	24 (18%)	96 (30%)	31 (30%)	0.04
Atrial fibrillation, n. (%)	131 (24%)	0 (0%)	29 (9%)	102 (100%)	<0.01
**Laboratory Values**					
eGFR, ml/min/1.73m^2	64 ± 24	76 ± 29	60.5 ± 22	60.5 ± 21.7	<0.01
eGFR <30, n. (%)	32 (6%)	5 (4%)	22 (7%)	5 (5%)	0.43
NT-proBNP, ng/l	288 (183 - 572)	232 (171 - 375)	283 (183 - 542)	454 (230 - 1011)	<0.01
CRP, mg/l	2.5 (1.2-5.2)	2.1 (0.9-4.7)	2.5 (1.2-5)	3.0 (1.6-5.4)	0.08
Troponin T, pg/ml	10.8 (7.2 - 12)	8.8 (5.7 - 10.8)	10.8 (7.8 - 11.7)	10.8 (8.5 - 14)	<0.01
**Echocardiographic Parameters**					
LV-EF, %	61 ± 6.6	60 ± 6	61 ± 6.8	61.5 ± 6.9	0.57
E/e'	10 ± 3.3	8 ± 1.9	10.6 ± 2.8	12.3 ± 3.7	<0.01
LV-EDV index, ml/m²	54.4 ± 18	60.7 ± 18.7	53 ± 17	50.7 ± 17.8	<0.01
LV-Mass index, g/m²	138 ± 40	143 ± 40.6	136.4 ± 38.9	136.7 ± 37.9	0.25
LA diameter index, mm/m²	24 ± 3.7	24 ± 3.1	23.3 ± 3.4	25 ± 3.4	<0.01
TR Vmax, m/s	2.5 ± 0.4	2.3 ± 0.3	2.4 ± 0.4	2.6 ± 0.4	<0.01
Moderate valvular disease, n. (%)	75 (14%)	9 (7%)	41 (13%)	25 (25%)	<0.01
**Events during follow-up**					
HF rehospitalization, n. (%)	140 (25%)	18 (14%)	78 (24%)	44 (43%)	<0.01
Average number of rehospitalizations, n.	1.78 ± 1.8	1.25 ± 1.6	1.77 ± 1.8	2.5 ± 2.0	<0.01
All-cause mortality, n. (%)	62 (11%)	8 (6%)	33 (10%)	21 (21%)	<0.01

NYHA Class = New York Heart Association Class (1-4), BMI = body mass index, CAD = coronary artery disease, HF = heart failure, Hx = history, FU = follow-up, eGFR = estimated glomerular filtration rate, LV-EF = left ventricular ejection fraction, LV-EDV = left ventricular enddiastolic volume, LA = left atrial, TR Vmax = peak velocity of tricuspid valve regurgitation in CW Doppler, TAPSE = tricuspid annular plane systolic excursion. Percentages equal or greater 0.5 were rounded to the larger integer.

Table S3. *Logistic Regression for HF-hospitalization in cohort of patients with HFpEF and overlapping conditions at presentation*.

	**Logistic Regression Model (univariate)**				**Logistic Regression Model (multivariable)**			
		95.0% CI for EXP(B)				95.0% CI for EXP(B)		
	EXP(B)	Lower	Upper	p-value	EXP(B)	Lower	Upper	p-value
Male sex	0.99	0.65	1.50	0.98	1.12	0.72	1.73	0.63
lnNTproBNP	1.07	0.85	1.34	0.55	1.22	0.88	1.69	0.23
NYHA-class	0.59	0.30	1.20	0.15	0.42	0.15	1.16	0.10
H_2FPEF high-risk	2.82	1.79	4.33	<0.01	2.70	1.65	4.41	<0.01

H_2FPEF high risk = H_2FPEF score equal or greater than 6 as binary variable, lnNT-pro-BNP = natural logarithm of the NT-pro-BNP at baseline, NYHA-Class = New York Heart association class (1-4), EXP(B) is considered equivalent to odds ratio.
Hosmer and Lemeshow Test p =0.44, Cox & Snell R Square p=0.05, Nagelkerke R Square p= 0.73,
Age, Afib, BMI and E/e' were not inputted due to significant co-linearity with H_2FPEF score.

Table S4. *Logistic Regression for HF-hospitalization in cohort of patients with isolated HFpEF*

	**Logistic Regression Model (univariate)**				**Logistic Regression Model (multivariable)**			
		95.0% CI for EXP(B)				95.0% CI for EXP(B)		
	EXP(B)	Lower	Upper	p-value	EXP(B)	Lower	Upper	p-value
Male Sex	0.78	0.49	1.24	0.30	1.09	0.67	1.79	0.72
lnNTproBNP	0.97	0.72	1.29	0.84	0.95	0.78	1.02	0.13
NYHA-Class	1.00	0.41	2.42	0.99	0.61	0.28	1.31	0.96
H_2FPEF high-risk	3.81	2.33	6.24	<0.01	5.19	2.95	9.10	<0.01

H_2FPEF high risk = H_2FPEF score equal or greater than 6 as binary variable, lnNT-pro-BNP = natural logarithm of the NT-pro-BNP at baseline, NYHA-Class = New York Heart association class (1-4), EXP(B) is considered equivalent to odds ratio.
Hosmer and Lemeshow Test p =0.12, Cox & Snell R Square p=0.07, Nagelkerke R Square p= 0.11,
Age, Afib, BMI and E/e' were not inputted due to significant co-linearity with H_2FPEF score.

Figure S1. *Classification of the overall cohort with detailed reason of hospitalization for the subgroup of HFpEF with overlapping conditions.*

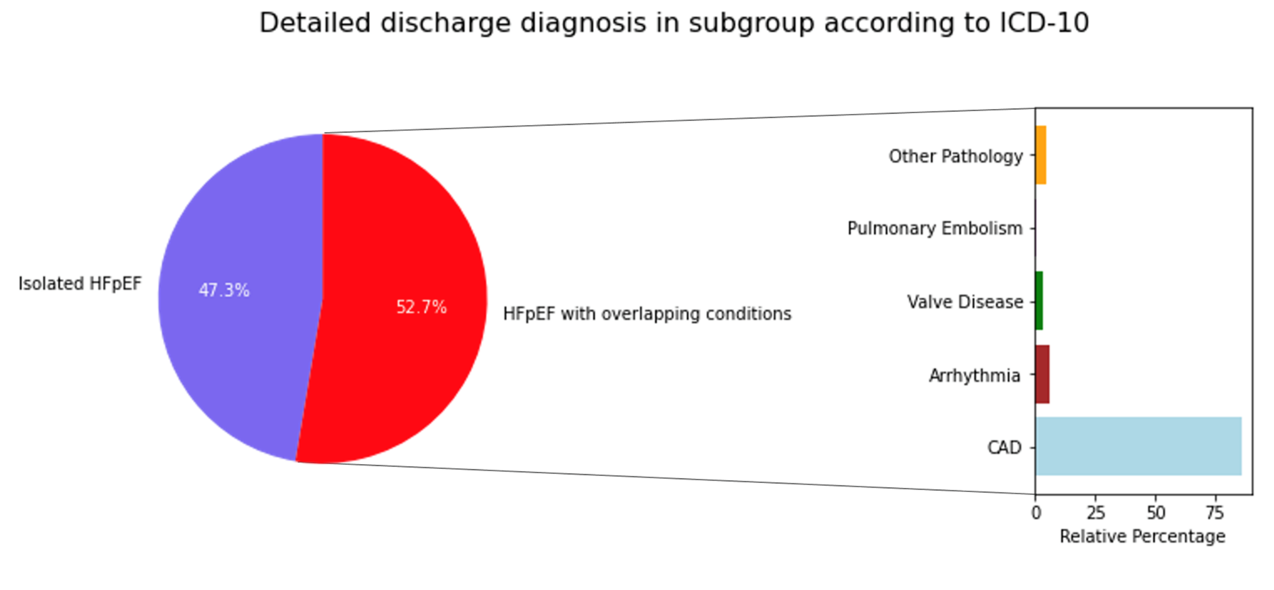

Legend: CAD = coronary artery disease, arrhythmia = brady- or tachyarrhythmia due to Afib, valve = moderate or severe valve disease. HFpEF = heart failure with preserved ejection fraction, ICD-10 = International Statistical Classification of Diseases and Related Health Problems (WHO Version)

Figure S2. *Detailed reasons of first rehospitalization.*

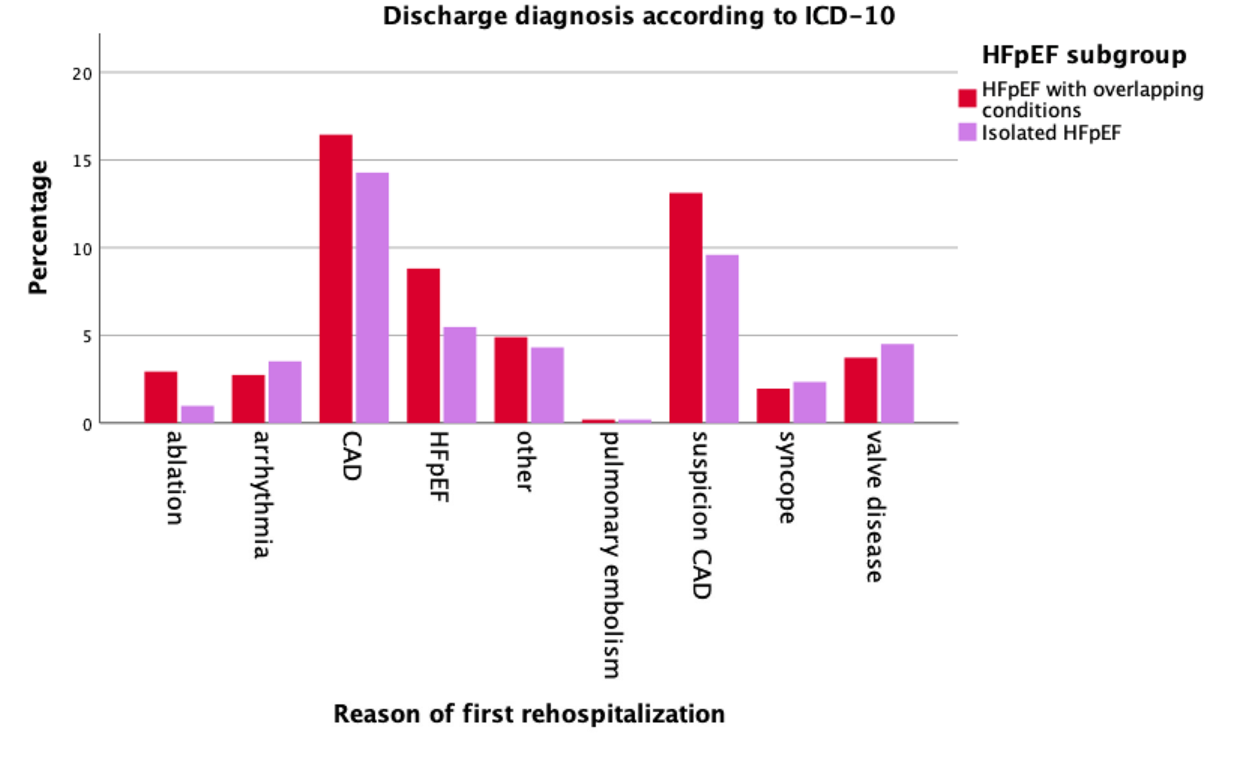

Legend: ablation = pulmonary vein isolation, arrhythmia = brady- or tachyarrhythmia due to Afib, CAD = coronary artery disease, ICD-10 = International Statistical Classification of Diseases and Related Health Problems (WHO Version), suspicion CAD = patients without obstructive CAD presenting with non-invasive signs of myocardial ischemia or typical anginal symptoms and high pretest probability of coronary artery disease, HFpEF = heart failure with preserved ejection fraction, valve disease = moderate or severe valve disease.

Figure S3. ROC curves for HFpEF hospitalization and death in overall cohort, CAD subgroup and Non-CAD subgroup.

<table>
<tr><th>ROC curve for HFpEF hospitalization in overall cohort</th><th>ROC curve for HFpEF hospitalization in CAD subgroup</th><th>ROC curve for HFpEF hospitalization in Non-CAD subgroup</th></tr>
<tr><td>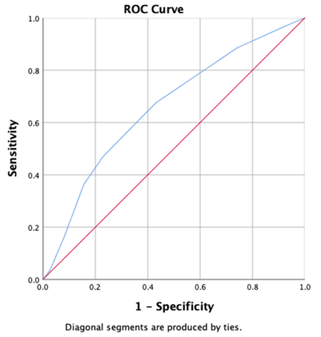
</td><td>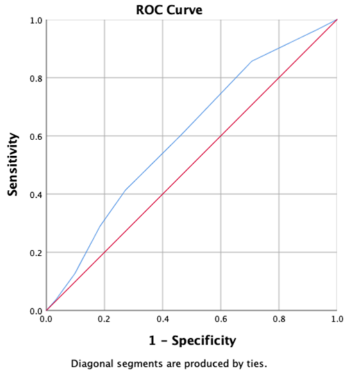
</td><td>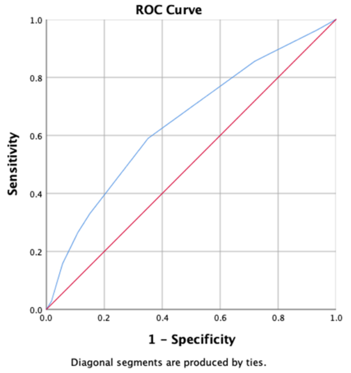
</td></tr>
<tr><td>AUC 0.63, CI 0.59-0.66, p<0.01</td><td>AUC 0.60, CI 0.56-0.65, p<0.01</td><td>AUC 0.65, CI 0.59-0.70, p<0.01</td></tr>
</table>

Figure S4. KM-estimates for isolated HFpEF and HFpEF with overlapping conditions in H_2FpEF score high risk group.

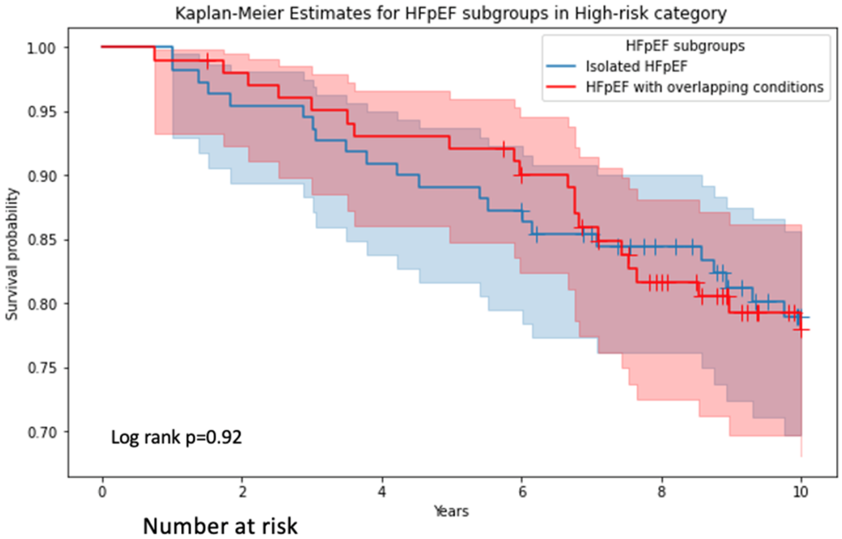

Number at risk

	2 years	4 years	6 years	8 years	10 years
**Isolated HFpEF**	105	100	96	82	78
**HFpEF with overlapping conditions**	99	94	90	74	65

Kaplan-Meier estimates for patients according to HFpEF with overlapping conditions and isolated HFpEF in high-risk H_2FPEF category. Ticks represent censored data. Legend: H_2FPEF = scoring system based on age, BMI, atrial fibrillation, pulmonary hypertension, elevated filling pressures, and arterial hypertension, High risk class is defined as a point-score ≥ 6.
